# *BrCWM* Mutation Disrupted Leaf Flattening in Chinese Cabbage (*Brassica rapa* L. ssp. *pekinensis*)

**DOI:** 10.3390/ijms24065225

**Published:** 2023-03-09

**Authors:** Yanji Wu, Yue Xin, Jiaqi Zou, Shengnan Huang, Che Wang, Hui Feng

**Affiliations:** Liaoning Key Laboratory of Genetics and Breeding for Cruciferous Vegetable Crops, College of Horticulture, Shenyang Agricultural University, Shenyang 110065, China

**Keywords:** Chinese cabbage, wrinkled leaves, cortical microtubule, gene cloning, mutant

## Abstract

Leaf flattening plays a vital role in the establishment of plant architecture, which is closely related to plant photosynthesis and, thus, influences the product yield and quality of Chinese cabbage. In this study, we used the doubled haploid line ‘FT’ of Chinese cabbage as the wild type for ethyl methanesulfonate (EMS) mutagenesis and obtained a mutant *cwm* with stably inherited compact and wrinkled leaves. Genetic analysis revealed that the mutated trait was controlled by a single recessive nuclear gene, *Brcwm*. *Brcwm* was preliminarily mapped to chromosome A07 based on bulked segregant RNA sequencing (BSR-seq) and fine-mapped to a 205.66 kb region containing 39 genes between Indel12 and Indel21 using SSR and Indel analysis. According to the whole-genome re-sequencing results, we found that there was only one nonsynonymous single nucleotide polymorphism (SNP) (C to T) within the target interval on exon 4 of *BraA07g021970.3C*, which resulted in a proline to serine amino acid substitution. The mutated trait co-segregated with the SNP. Quantitative reverse transcriptase polymerase chain reaction (qRT-PCR) revealed that *BraA07g021970.3C* expression was dramatically higher in ‘FT’ leaves than that in *cwm* leaves. *BraA07g021970.3C* is homologous to *AT3G55000* encoding a protein related to cortical microtubule organization. A similar phenotype of dwarfism and wrinkled leaves was observed in the recessive homozygous mutant *cwm-f1* of AT3G55000, and its T3 transgenic lines were restored to the *Arabidopsis* wild-type phenotype through ectopic overexpression of *BraA07g021970.3C*. These results verified that *BraA07g021970.3C* was the target gene essential for leaf flattening in Chinese cabbage.

## 1. Introduction

Leaves are the most important photosynthetic organs in plants, and their morphology and functionality directly determine crop productivity by affecting plant architecture, respiration, transpiration, and nutrient absorption [1,2]. For most plants, their leaves would develop into flat laminas, which is a natural adaptation to enlarge the area for capturing light. Therefore, leaf flattening is an important agronomic trait for crop breeding.

Leaf morphological development is a complicated process regulated by transcription factors, gene expression, and environmental factors [3]. Several mutants related to leaf flatness have been identified and their mutant genes have been mapped and cloned. NJAU5737, with up curled and slightly crinkled leaves, has been identified in *Brassica napus*. On chromosome A05, an 83.19 kb interval contained the dominant locus (*BnUC2*) that controls the trait. The candidate gene *BnaA05g16700D* encodes a regulatory protein for auxin signal transcription [4]. Two curly leaf mutants, cul-1 and cul-2 have been identified in cucumber. The mutant gene is mapped to chromosome 6 in a 128 kb region. As the candidate gene, *CsPHB* encodes the HD-ZIP III transcription factor [5]. Research has shown that in the RL-D mutant, a single dominant gene controls the leaf-rolling trait and is located on rice chromosome 3 within a 743 kb region. The candidate gene identified is *Os03g0395100*, which encodes a protein phosphatase [6]. *lad1* is a rolling-leaf mutant of rice. The mutant gene *KAN1* maps on chromosome 9 within 34 kb intervals. The candidate gene *KAN1* interacts with ARF3, ARF7, and ARF15, playing a conservative role during leaf development [7]. These studies provide valuable genetic resources and references for elucidating the mechanisms underlying leaf morphology and plant development.

Plant microtubules (MTs) are subcellular nanotubes composed of α-tubulin and β-tubulin that participate in cell mitosis, intracellular material transport, cell wall construction, morphogenesis, and polarity establishment [8,9,10,11,12]. During the cell cycle, MTs are present in four distinct arrays: cortical microtubules (CMTs), preprophase band (PPB), spindle, and phragmoplast. In response to microtubule arrays, microtubule-associated proteins (MAPs) bind to microtubes and modulate their functions. Recent studies have shown that MAPs, such as MOR1, TRM, FASS, TON1, kinesins, CLASP, and TAN1, affect microtubule arrays and plant development [13,14,15,16,17]. Among these genes, TON1 has been reported to be a cortical microtubule-related protein. Studies have shown that the disruption of *TON1* leads to the disappearance of PPB, and the *TON1* deletion mutant displays irregular cell elongation and division planes [15,16]. As a matter of fact, the role of TON1 homologous proteins in the plant development of horticultural crops remains largely unknown.

Chinese cabbage is widely cultivated in Eastern Asia. As a vital vegetable crop, its leaves are not only the photosynthetic apparatus but also the main product organ. Leaf morphology directly affects yield and commodity value. In this study, a compact and wrinkled mutant *cwm* was obtained from an ethyl methanesulfonate (EMS) mutagenesis population of Chinese cabbage, and the molecular mechanism of leaf flattening was explored. *BraA07g021970.3C* was referred to as the candidate gene for mutation traits using bulked segregant RNA sequencing (BSR-seq) and whole-genome re-sequencing. The *Arabidopsis cwm-f1* mutant with wrinkled leaves restored its normal phenotype by ectopic overexpression of *BrCWM*.

## 2. Results

### 2.1. Morphological Characterization of the Mutant Cwm

**Figure 1:**
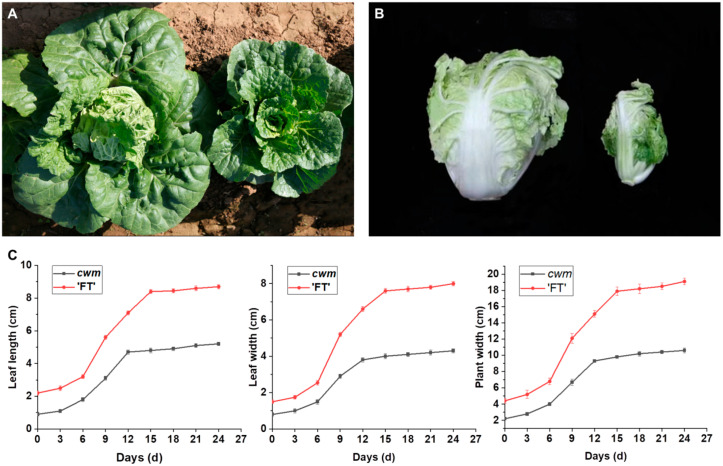
Phenotypic characterization of ‘FT’ and *cwm*. (**A**) The phenotypic characterization of ‘FT’ (left) and *cwm* (right) at the heading stage. (**B**) The identification of leafy heads in ‘FT’ (left) and *cwm* (right). (**C**) The growth curves of *cwm* and ‘FT’ at the seedling stage.

*cwm* exhibited a compact plant with wrinkled leaves in contrast to the wild type ‘FT’ (Figure 1A). The mutant leafy head was remarkably smaller than ‘FT’ (Figure 1B; Table S5). Based on the growth curves, leaf length, leaf width, and plant width increased more slowly in *cwm* following the appearance of the third true leaf (Figure 1C).

**Figure 2:**
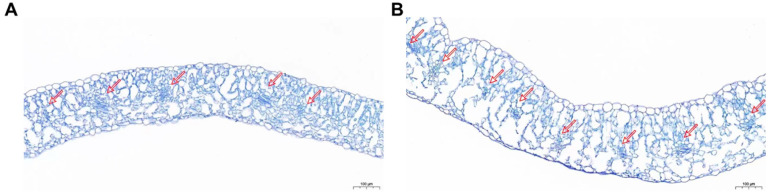
Paraffin section of the leaf transverse section. Scale bar = 100 μm. (**A**) Leaf transverse sections of ‘FT’. (**B**) Leaf transverse sections of *cwm*. The red arrows indicated the vascular bundles in the leaf mesophyll.

To further characterize the leaf morphology, the paraffin sections were performed. In *cwm*, there were more vascular bundles and thicker leaves, and in both spongy mesophyll and palisade mesophyll, the lacunas were larger, compared to ‘FT’. (Figure 2).

### 2.2. Inheritance of the Mutated Traits

The results of the genetic analysis are listed in Table 1. All F_1_ plants exhibited the wild-type phenotype, indicating that the mutant traits were controlled by a recessive gene. Among the 250 F_2_ individuals, 184 plants exhibited wild-type traits, while the rest exhibited mutant traits, in consonance with the segregation ratio of 3:1 (χ^2^  =  0.192). Forty plants from BC_1_ (F_1_ × ‘FT’) were all same as the ‘FT’ phenotype. Among the BC_1_ (F_1_ × *cwm*) plants, 19 and 21 showed the ‘FT’ phenotype and *cwm* phenotype, respectively, which conformed to a segregation ratio of 1:1 (χ^2^ = 0.100). Taken together, these mutant traits were controlled by a single recessive nuclear gene, *Brcwm*.

### 2.3. Preliminary Mapping of Brcwm Using BSR-Seq

**Figure 3:**
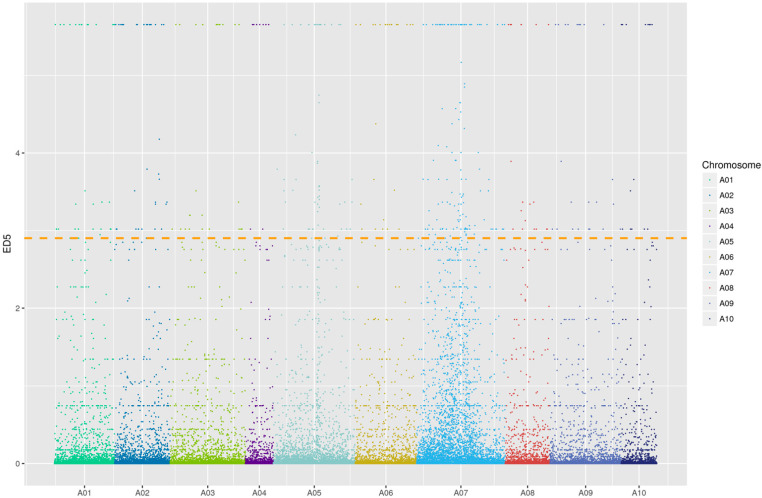
Map of ED^5^ distribution on chromosomes. The width of each color on the X-axis represented the quantity of different SNV sites on each chromosome. The Y-axis represented the ED^5^ values of each differential SNV site. The horizontal line referred to the threshold of the top 1%.

*Brcwm* was preliminarily mapped using BSR-Seq. Among the 39,346,988 and 41,518,202 clean reads within the mutation pool and wild-type pool, respectively, the GC content and Q30 (the base quality value was 30, and the error rate was 0.1%) percentage revealed that the sequencing data were extremely precise (Table 2). The total mapping ratio from clean reads to Brara_Chiifu_V3.0 (http://brassicadb.cn, accessed on 5 May 2022) was 0.81 > 0.7, suggesting that there were high sequence similarities between them (Table 2). Using the analysis of the single-nucleotide variant (SNV) difference and the value of Euclidean distance^5^ (ED^5^), the distribution of ED^5^ values on the chromosomes was illustrated (Figure 3). The *Brcwm* loci were located in four target candidate regions: A05 24538973-26614010, A07 10076460-11983310, A07 13806342-15548454, and A07 17469944-20228803 (Table 3).

**Figure 4:**
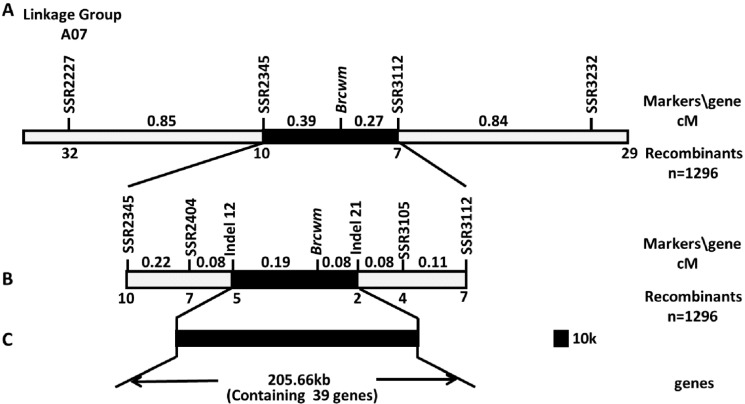
Mapping of the candidate gene. (**A**) Preliminary mapping of *Brcwm* based on the SSR primers screening recombinants. (**B**) Fine mapping of *Brcwm* based on the SSR and InDel primers screening recombinants. (**C**) Candidate gene analysis of *Brcwm*. In total, 1296 F_2_ recessive individuals with wrinkled leaves phenotype were screened to construct the linkage map of chromosome A07. The figures (cM) above chromosome A07 stood for the genetic distance. The figures under chromosome A07 stood for the recombinants.

To further narrow the range of *Brcwm* locations, a total of 15 pairs of polymorphic primers were screened out from 98 designed SSR primers. Three hundred recessive homozygous F_2_ individuals were used to validate that SSR2227 and SSR3232 on A07 were closely linked to *Brcwm* (Figure S1, Table S1). Thus, *Brcwm* was mapped on chromosome A07 between markers SSR2227 and SSR3232, with 1.23 cM and 1.11 cM genetic distances, respectively (Figure 4A).

### 2.4. Fine Mapping of Brcwm

To identify the mutation loci, 70 newly designed SSR and Indel markers were developed between markers SSR2227 and SSR3232. SSR2345, SSR2404, Indel12, Indel21, SSR3105, and SSR3112 with polymorphic markers were screened. Based on the mapping population of 1296 F_2_ plants with the mutant phenotype, *Brcwm* was mapped between Indel12 and Indel21 according to the label of recombinants, and the physical distance was about 205.66 kb (Figure 4B,C).

### 2.5. Candidate Gene Screening for Cwm using Whole Genome Re-Sequencing

Within the candidate interval, there were 39 genes referring to the *Brassica* database (http://brassicadb.cn/, accessed on 5 May 2022) (Figure 4C). Whole-genome re-sequencing of the mapping parents was conducted, and there was only one nonsynonymous SNP(C-T) on the exons within the mapping region. Because the SNP was on the fourth exon of *BraA07g021970.3C*, we identified *BraA07g021970.3C* as the candidate gene for *Brcwm*. According to genome annotations, *BrCWM* is homologous to *AT3G55000*, which encodes a protein related to cortical microtubule organization.

### 2.6. Clone Sequencing and Co-Segregation Analysis

**Figure 5:**
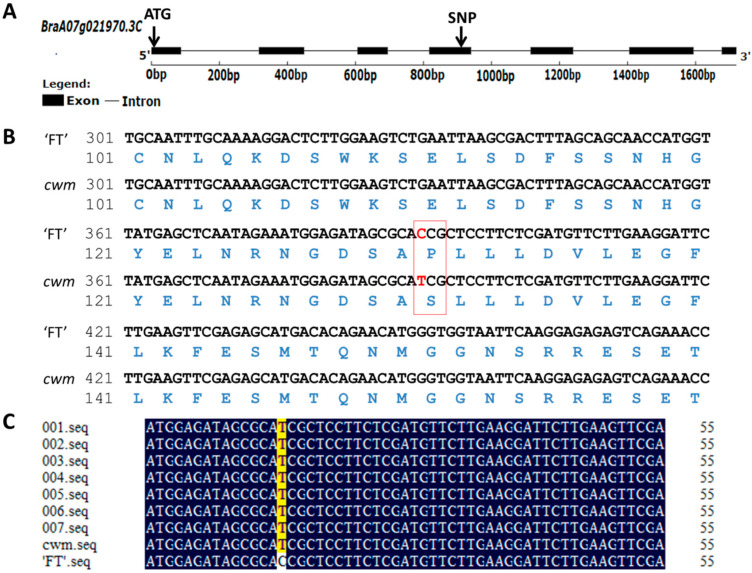
Gene structure, the alignment of gene sequence and amino acid sequence, and the co-segregation analysis. (**A**) Structure of *BraA07g21970.3C* with exon (the black square), intron (the black solid line), and nonsynonymous SNP and initiator codons (ATG). (**B**) Sequence alignments of partial amino acid and nucleotide of *BraA07g021970.3C* in ‘FT’ and *cwm*. The codons and amino acids in the red box resulted in the mutation. The nonsynonymous SNPs were shown in red color. (**C**) Sequence alignments of the *BraA07g021970.3C* nucleotides in seven F_2_ recombinants, *cwm,* and ‘FT’. The seven F_2_ recombinants were screened by markers indel12 and indel21.

Cloning and sequencing were conducted to confirm the variation in gene sequence between ‘FT’ and *cwm*. The results showed that a C to T substitution occurred in *cwm*, resulting in an amino acid change from Pro to Ser (Figure 5A,B and Figure S2).

The mutation sites in seven recombinants identified by Indel12 and Indel21 were cloned, which were the same as those in *cwm*, indicating that this SNP co-segregated with the leaf wrinkled phenotype (Figure 5C). Thus, *BraA07g021970.3C* was confirmed as the causal gene for *cwm*.

### 2.7. Expression Analysis of BrCWM

**Figure 6:**
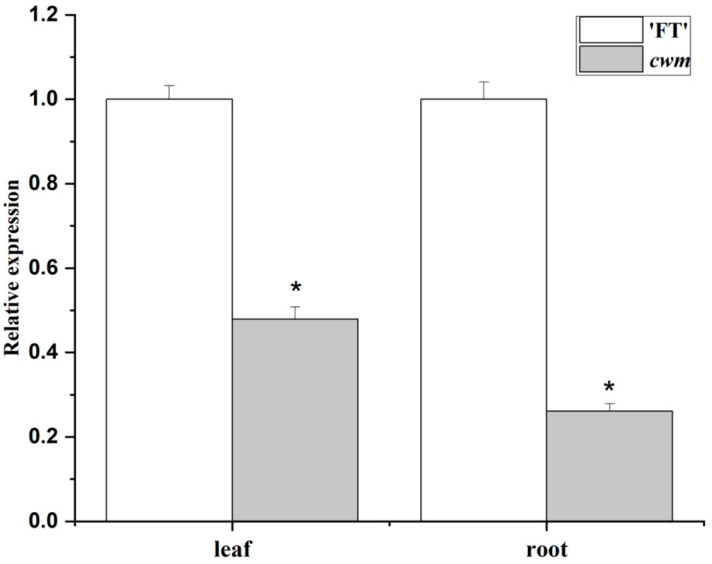
Expression levels of *BraA07g021970.3C* in leaves and roots were detected using qRT-PCR. Asterisks indicated statistical significance (Student’s *t*-test, * *p* < 0.05).

To confirm whether there was a difference in *BraA07g021970.3C* expression levels between the wild-type and mutant, qRT-PCR was performed. The data demonstrated that whether in the leaves or roots, *BraA07g021970.3C* expressions were remarkably lower in *cwm* than that in ‘FT’ (Figure 6).

### 2.8. Bioinformatic Analysis of BrCWM

**Figure 7:**
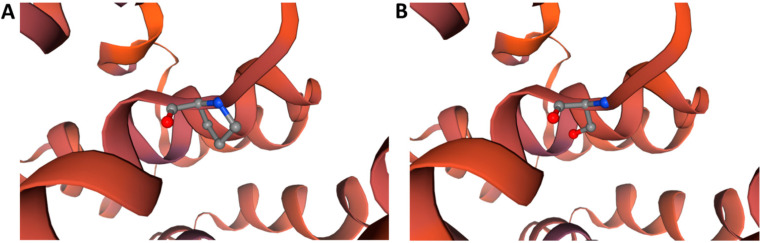
Protein tertiary structure analysis. (**A**) Tertiary structure of BrCWM. (**B**) Tertiary structure of the mutated BrCWM.

**Figure 8:**
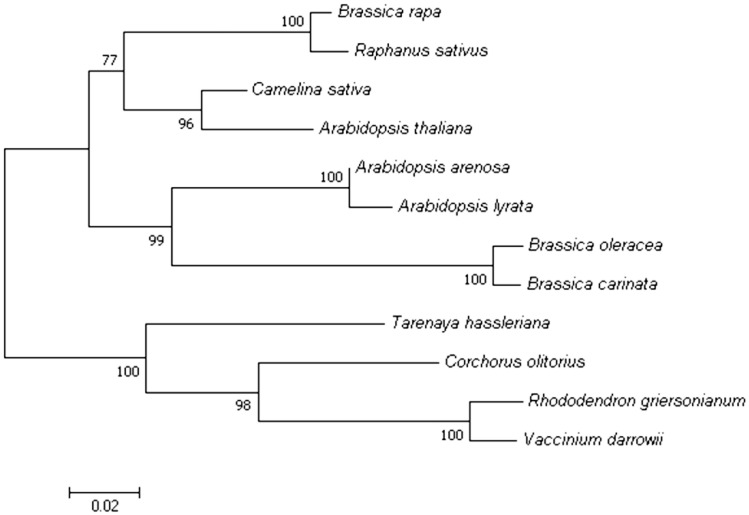
Phylogenetic tree analysis of BrCWM. The branch length which indicated evolutionary distance was drawn to scale.

To further elucidate the potential function of BrCWM, a series of bioinformatic analyses were performed. The result of TMHMM-2.0 forecasted that BrCWM has no transmembrane domain and is most likely a globular protein (Figure S3). The SWISS-MODEL software predicted that the substitution of amino acid residues in the mutation site resulted in tertiary structural changes in BrCWM, but there was no alteration in the holistic spatial structure (Figure 7). To clarify the possible phylogenetic relationship between BrCWM and other species, a phylogenetic tree was constructed using MEGA7 and NCBI BLAST. Eleven homologous proteins of BrCWM have been selected from other species. The results showed that the homology between BrCWM and *Raphanus sativus* TONNEAU 1a-like protein was the highest, reaching 92.37% (Figure 8).

### 2.9. Transgenic Functional Verification of BrCWM

**Figure 9:**
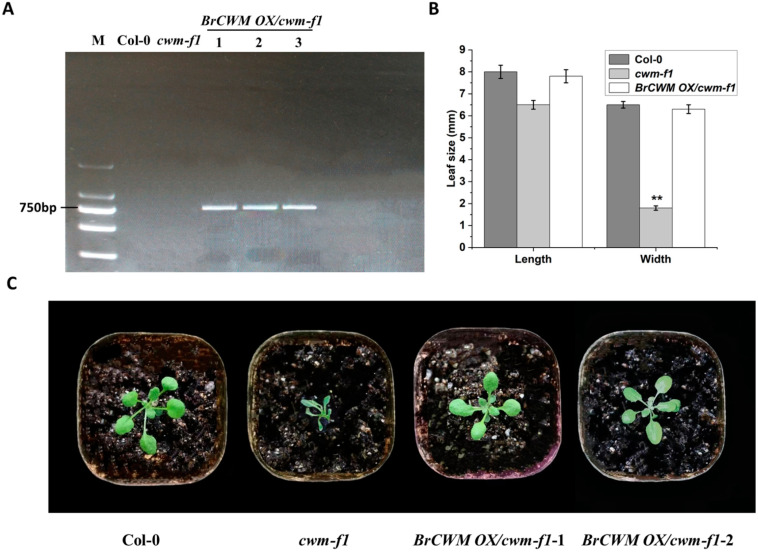
Transgenic functional verification of *BrCWM*. (**A**) Identification of the transgenic lines based on the PCR amplification of the cDNA sequence of *BrCWM* in *Arabidopsis*. M referred to molecular size markers; *BrCWM OX/cwm-f1* referred to transgenic *Arabidopsis* plants expressing *BrCWM*. (**B**) Statistical analysis of leaf length and width of Col-0, *cwm-f1*, and *BrCWM OX/cwm-f1*. Values are the means ± SD (*n* = 15 seedlings). Statistically significant differences were calculated using Student’s *t*-tests (** *p* < 0.01). (**C**) Morphology of Col-0, *cwm-f1*, *BrCWM OX/cwm-f1*-1, and *BrCWM OX/cwm-f1*-2 in a 2-week-old.

*BrCWM* is homologous to *AT3G55000*. Mutation of *AT3G55000* in *Arabidopsis ton1* resulted in the dysfunction of the cortical cytoskeleton and the absence of the preprophase band of microtubules, and the mutant plants were malformed [18]. In this study, we introduced *Arabidopsis cwm-f1* (CS2017348), an EMS-induced mutant that lacks the function of AT3G55000. The *cwm-f1* mutant exhibited wrinkled leaves and a dwarfing phenotype (Figure 9C). To verify whether *BrCWM* shares a similar function with *Arabidopsis AT3G55000*, we implemented an ectopic overexpression assay for *cwm-f1* by introducing 35S: BrCWM: GFP. Transgenic lines (homozygous T3-positive lines) were identified with PCR using primer W-1 (Figure 9A; Table S6). Col-0 and transgenic plants (*BrCWM OX/cwm-f1*) exhibited no significant morphological differences, whereas the leaf width of *cwm-f1* plants was remarkably narrower, resulting in a wrinkled phenotype (Figure 9B,C). These results indicated that *BrCWM* is functional in *Arabidopsis*.

## 3. Discussion

In this research, the *cwm* mutant with wrinkled leaves was obtained with EMS mutagenesis from a DH line ‘FT’ in Chinese cabbage. A recessive nuclear gene, *Brcwm*, which governed the mutant phenotype, was fine-mapped using BSR-seq and whole-genome re-sequencing. An SNP transition (C to T) in exon 4 of *BrCWM* caused an amino acid change from Pro to Ser, which was responsible for the wrinkled leaves in the mutant. Transgenic *BrCWM* rescued the mutant phenotype with wrinkled leaves in *Arabidopsis cwm-f1*. In conclusion, as a novel target gene, *BrCWM* is essential for leaf-flattening. These findings provide new insights into the potential molecular mechanisms of leaf morphogenesis and plant development in Chinese cabbage.

Moderately wrinkled leaves and compact plants can increase planting density and reduce leaf transpiration, resulting in improved photosynthetic efficiency and increased yield [19]. Leaf initiation, outgrowth, expansion, maturation, and polarity can affect leaf flattening to a certain extent. *KNOX1*, *CLV*, and *PIN1* influence leaf initiation, thereby affecting leaf shape [20,21,22]. *YABBY*, *HD-ZIPIII*, *KANADI,* and *AUXIN RESPONSE FACTOR* (*ARF*) gene families have an influence on leaf adaxial or abaxial polarity specification [23,24,25,26]. The *GRF* gene family acts as a regulator of cell proliferation and regulates the size and shape of the leaves [27]. In addition, *WOX* and *YUCCA* gene families are essential for leaf development [28,29].

Leaf flattening can be influenced by the known genes associated with leaf morphology. For example, the mutation of *NRL1*, which encodes a cellulose synthase-like protein, results in semi-rolled leaves in rice [30]. *REL* encodes a protein with DUF630 and DUF632 domains that give rise to leaf rolling in rice [31]. The mutation of *BnaA03.IAA7* blocked auxin signaling, resulting in crinkle leaves and dwarfing plants [32]. Our research found that as the candidate gene of leaf wrinkled mutation, *BraA07g021970.3C* encodes a homolog of TON1, which is relevant to cortical microtubule (CMT) organization. Previous studies have shown that CMTs influence cellulose microfibril orientation, which in turn determines cell morphology [33]. Cell wall development is regulated by interactions between several endomembrane systems and CMTs [34]. TON1 has been shown to interact with *Arabidopsis* CENTRIN [16]. Moreover, TRM1 interacts with TON1 in *Arabidopsis* [35]. In *Arabidopsis* and tomato leaves, the process of leaf flattening depends on the arrangement of CMTs parallel to the adaxial–abaxial axis of the leaf in the direction of maximum stress [36]. Changes in cellulose content and cell walls can affect rice leaf flattening [37]. Here, we found more vascular bundles in *cwm* with the paraffin section observation (Figure 2). Therefore, we hypothesized that more vascular bundles result in a wrinkled phenotype. The downregulation of *BrCWM* expression in *cwm* leaves (Figure 6) might disrupt the CMT-mediated orientation of cellulose microfibrils and affect the formation of vascular bundles, which lead to more vascular bundles, ultimately leading to a wrinkled leaf phenotype.

Previous studies have indicated that the *TON1* deletion mutant changed the orientation of the symmetric divisions, resulting in a shorter root length than the normal plant, and the mutant plants were dwarf and malformed [15,16]. Our study demonstrated that the root length was remarkably shorter in the mutant *cwm*, and the expression of *BrCWM* was significantly lower in the roots of *cwm* during the growth period (Figures S4 and S5). Our results are consistent with those of previous studies. Plant roots can absorb water and inorganic salts, and there is a correlation between roots and leaves, which together determine the growth and development of plants [38]. Thus, we hypothesized that the dysfunction of *BrCWM* results in the irregular orientation of the symmetric divisions in the roots of *cwm*, further inhibiting root development and its ability to absorb nutrients and water. Consequently, the aerial part had fewer nutrients and water, leading to the wrinkled leaf phenotype in *cwm*.

Our study verified that the mutation of *BrCWM* led to wrinkled leaves. This is the first study to show that cortical microtubule-related proteins may be involved in leaf flattening in Chinese cabbage. These results provide an entirely new perspective for exploring the molecular mechanisms of leaf flattening in Chinese cabbage.

## 4. Materials and Methods

### 4.1. Plant Materials and Growth Conditions

A Chinese cabbage doubled haploid (DH) line ‘FT’ was designated as wild type. The *cwm* mutant was generated using 0.8% EMS mutagenesis. The ‘701’ was a DH line of Chinese cabbage with a distant genetic background from ‘FT’. They were grown in plastic greenhouses at the Shenyang Agricultural University, Shenyang, China, in 2018.

Wild-type *Arabidopsis* (Col-0) was preserved at the Shenyang Agricultural University. *cwm-f1* (CS2107348) was obtained from Arabidopsis Biological Resource Center (ABRC, Columbus, OH, USA). The plants were grown in a culture room with a cycle of 16 h light/8 h dark and 80% relative humidity at 22 °C.

### 4.2. Growth Determination

Five *cwm* and ‘FT’ plants with consistent growth were randomly selected for investigation. The leaf length, leaf width, and plant width were determined every 3 d for 24 d, starting from the appearance of the third true leaf. At the end of the heading stage, the length, width, and weight of the leafy heads were measured. The average value of the data for each group was used for plotting.

### 4.3. Cytological Observation

To identify whether the phenotype of a wrinkled leaf was influenced by the change in cell morphology, the mesophyll cells of *cwm* and ‘FT’ were observed with a paraffin section. Several 2 cm * 1 cm slices were cut from the same parts of the sixth true leaf of *cwm* and ‘FT’, which were immediately fixed in FAA containing 50% ethanol, 5% glacial acetic, and 10% formalin for 24 h at 25 °C. Subsequently, dehydration was performed using gradient alcohol concentrations (50–100%). The samples were then permeated with xylene. The samples were embedded in paraffin. A microtome (LeicaRM2016, Wetzlar, Germany) was used to treat the paraffin sections, and an optical microscope (Nikon ECLIPSE 80i, Tokyo, Japan) was used to observe the stained samples.

### 4.4. Genetic Analysis

The F_1_, F_2_, and BC_1_ generations were created to verify the genetic characteristics of the wrinkled leaf phenotype. ‘FT’ and *cwm* were crossed to generate F_1_. F_1_ plants were self-pollinated to generate the F_2_ population. F_1_ and the parent of *cwm* or ‘FT’ were crossed to produce BC_1_ generations. The Chi-square (χ2) test was conducted to analyze the segregation ratios of the BC_1_ and F_2_ populations.

### 4.5. BSR-Sequencing Analysis

The F_2_ segregating population for BSR-Seq and mapping was derived from the crossing of *cwm* and ‘701’. The leaves of 35 individuals with mutant and normal traits were sampled from the F_2_ population and divided into two mixed pools. TRIzol Reagent (Invitrogen, Carlsbad, CA, USA) and an RNeasy Mini Kit (Qiagen, Beijing, China) were used to extract total RNA from each sample. Total RNA quality was determined using an Agilent bioanalyzer 2100 (Agilent Technologies, Palo Alto, CA, USA). Library construction was performed using a NEBNext Ultra RNA Library Prep Kit (Illumina, San Diego, CA, USA).

Transcriptome sequencing of the two pools was conducted using the Illumina HiSeq platform GENEWIZ (Suzhou, China). Cutadapt (version 1.9.1) was used to pretreat the raw reads, filter low-quality data, and eliminate pollution and connector sequences. The comparison results were compiled using Samtools (version 1.3.1) to obtain the possible single nucleotide variant (SNV) results that were annotated using Annovar software. According to the top 1% of the Euclidean distance^5^ (ED^5^) value of the differential SNV and the distribution map of the differential SNV on chromosomes, chromosome segments related to the traits were located.

### 4.6. DNA Extraction, Primer Design, and PCR

The genomic DNA of the leaves or roots was extracted using the cetyltrimethylammonium bromide (CTAB) method. The DNA concentration was regulated to approximately 30 ng/μL.

Initially, SSR markers were designed and polymorphic markers were screened using polyacrylamide gel electrophoresis between *cwm* and ‘701’. Other SSR markers and Indel markers were designed to narrow the mapping range using Primer Premier software (version 5.0) and synthesized in Sangon (Shanghai, China) (Table S1).

The PCR was conducted in a 10 μL volume and started with a step of 95 °C for 5 min, followed by 35 cycles of 95 °C for 30 s and 56 °C for 30 s and with a final extension at 72 °C for 5 min.

### 4.7. Whole-Genome Re-Sequencing

A library with 400bp inserted fragments was constructed and paired-end (PE) sequencing of the library was carried out using Next Generation Sequencing (NGS). High-quality data were obtained with the removal of joint contamination using Adapter Removal [39], quality filtering, and length filtering. Using mapping between the reference genome (http://brassicadb.cn/, accessed on 5 May 2022) and high-quality data using BWA-MEM, single nucleotide polymorphism (SNP), insertion and deletion (INEDL), copy number variation (CNV), and structural variation of the chromosome (SV) were screened. GATK [40] and ANNOVAR [41] were used for SNP detection and annotation, respectively.

### 4.8. Clone Sequencing

To verify the SNP mutation in *BrCWM*, the full-length sequence of *BrCWM* was obtained using cloning and sequencing. The cloning primer, C-1, was designed as shown in Table S2. The PCR products were linked using the Biorun Seamless Cloning Kit(#RDA01) (Biorun, Wuhan, China) and then transformed into competent cells (Biorun, Wuhan, China). The positive clones were sequenced using GENEWIZ (Tianjin, China), and the sequences were aligned using DNAMAN (version 5.2.9).

### 4.9. Quantitative Reverse Transcriptase PCR (qRT-PCR)

To confirm the candidate gene expression pattern, a plant total RNA extraction kit (Tiangen, Beijing, China) was used to extract total RNA from roots and leaves at the fifth true leaf stage of ‘FT’ and *cwm* seedlings. The FastQuant RT Super Mix (Tiangen, Beijing, China) was used to synthesize cDNA. The primer RT-1 designed by Primer Premier 5.0 (Table S3) and the SYBR Green PCR Master Mix (Takara Bio Inc., Kusatsu, Japan) were used for qRT-PCR. The data were analyzed using QuantStudio^TM^ Real-Time PCR software. The program and reaction volume of the qRT-PCR were described previously [42].

### 4.10. Bioinformatic Analysis

TMHMM-2.0 (https://www.cbs.dtu.dk/services/TMHMM-2.0/, accessed on 5 May 2022) was used to predict the transmembrane domain of BrCWM. The protein tertiary structure of BrCWM was predicted using SWISS-MODEL software (https://swissmodel.expasy.org/, accessed on 5 May 2022). The phylogenetic tree of BrCWM was established using MEGA7.0.26 (https://www.megasoftware.net, accessed on 5 May 2022).

### 4.11. Vector Construction and Arabidopsis Genetic Transformation

Homologous recombination and Golden Gate seamless cloning [43] were used for vector construction. The sequence of *BrCWM* was amplified from genomic DNA using the V-1 primer, as shown in Table S4, and recombined with pBWA(V)HS-ccdb-GFP. Then, 35S: BrCWM: GFP was introduced into *Arabidopsis* mutant *cwm-f1* with Agrobacterium-mediated genetic transformation. The inflorescences were dipped in bacterial solution for 2–3 s, then kept at humidity >90% using membrane sealing and incubated them in dark at 25 °C for 24 h. The impregnation cycle was seven days, with three times in total. The seeds were collected and sown. Positive transgenic plants were screened and raised to the 6–8th true leaf stage used for trait identification.

## Figures and Tables

**Table 1:** Genetic analysis of the wrinkled leaves phenotype in mutant *cwm*.

Generation	Normal Leaves	Wrinkled Leaves	Segregation Ratio	Expected Ratio	χ^2^
P_1_ (FT)	50	0			
P_2_ (*cwm*)	0	50			
P_1_ × P_2_	35	0			
P_2_ × P_1_	35	0			
(P_1_ × P_2_) × P_1_	40	0			
(P_1_ × P_2_) × P_2_	19	21	1.167:1	1:1	0.100
F_2_	184	66	2.787:1	3:1	0.192

**Table 2:** Summary of clean Illumina RNA-Seq reads from ‘FT’ and *cwm* samples.

Sample	Total Raw Reads	Total Clean Reads	Total Mapping	Total Mapping Ratio	Q30 (%)	GC Content (%)
‘FT’	39,548,850	39,346,988	31,994,128	0.81	95.23	48.22
*cwm*	41,723,712	41,518,202	33,960,896	0.81	95.23	48.30

**Table 3:** Localization of chromosome regions associated with the mutant traits.

Chromosome	Start Site	End Site	SNV Count	Target Length
A05	24,538,973	26,614,010	25	2,075,037
A07	10,076,460	11,983,310	20	1,906,850
A07	13,806,342	15,548,454	28	1,742,112
A07	17,469,944	20,228,803	57	2,758,859

## Data Availability

The raw sequence data of the BSR-Seq have been uploaded to the Sequence Read Archive at NCBI under the accession number SRR23004873 and SRR23004874. The raw sequence data of the whole genome re-sequencing have been uploaded to the Sequence Read Archive at NCBI under the accession number SRR23004840.

## Supplementary Materials

The following supporting information can be downloaded at: https://www.mdpi.com/article/10.3390/ijms24065225/s1.
