# Supplementary material for: BrCWM Mutation Disrupted Leaf Flattening in Chinese Cabbage (Brassica rapa L. ssp. pekinensis)
Source: Int J Mol Sci. 2023 Mar 9;24(6):5225. doi: 10.3390/ijms24065225 (PMC10049106; doi:10.3390/ijms24065225)
Supplement: Supplementary file 1 [file ijms-24-05225-s001.zip › ijms-2238408-supplementary.docx]

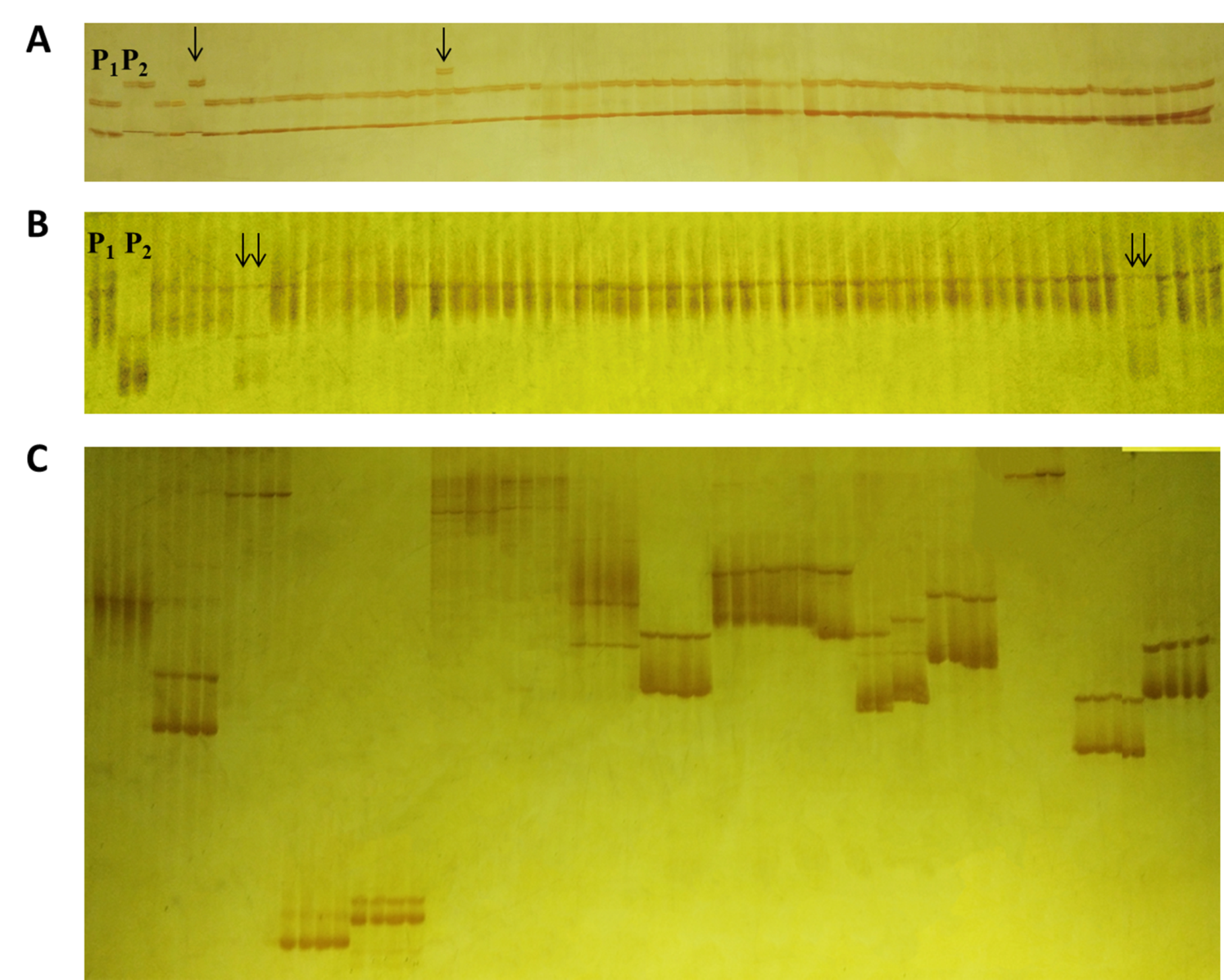


**Supplementary Figure S1.** Screening of molecular markers closely linked to *Brcwm*. (**A**) Recombinants with SSR3232 in the mapping population; (**B**) Recombinants with SSR2227 in the mapping population; (**C**) Screening of polymorphic SSR markers between the two parents. P_1_: mutant *cwm.* P_2_: DH line ‘701’. Arrows represented the recombinants.


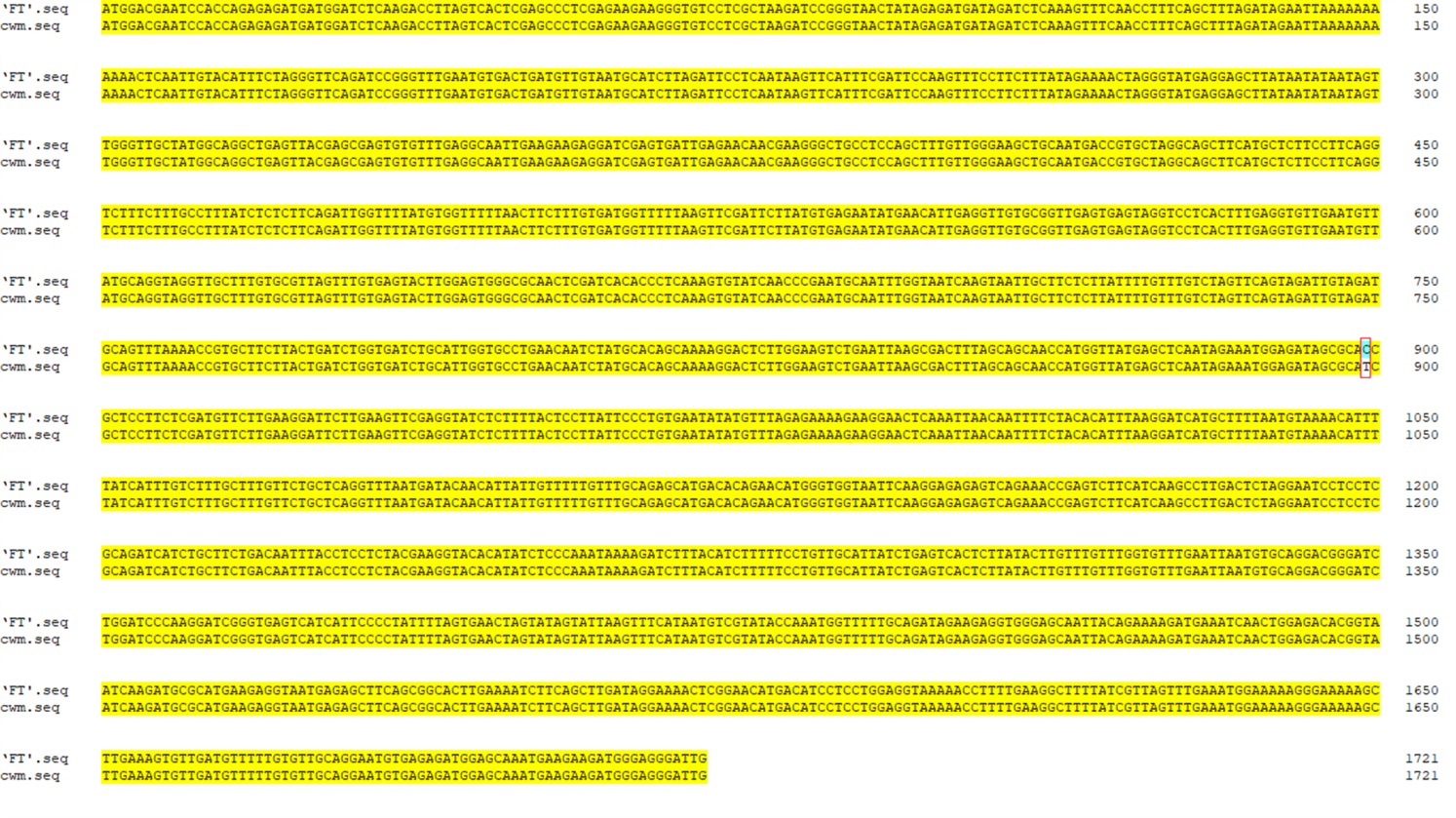


**Supplementary Figure S2.** Alignment of the nucleotide sequences of *BraA01g021970.3C* from wild-type ‘FT’ and mutant *cwm*. The difference between them was shown in the red frame.


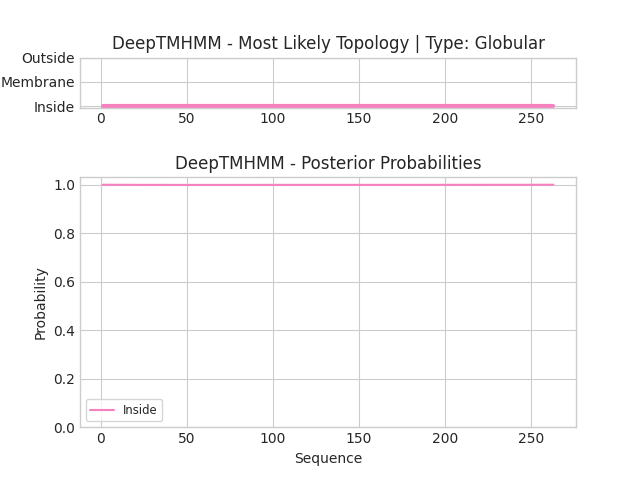


**Supplementary Figure S3.** The transmembrane domain prediction of BrCWM by the TMHMM-2.0 software.


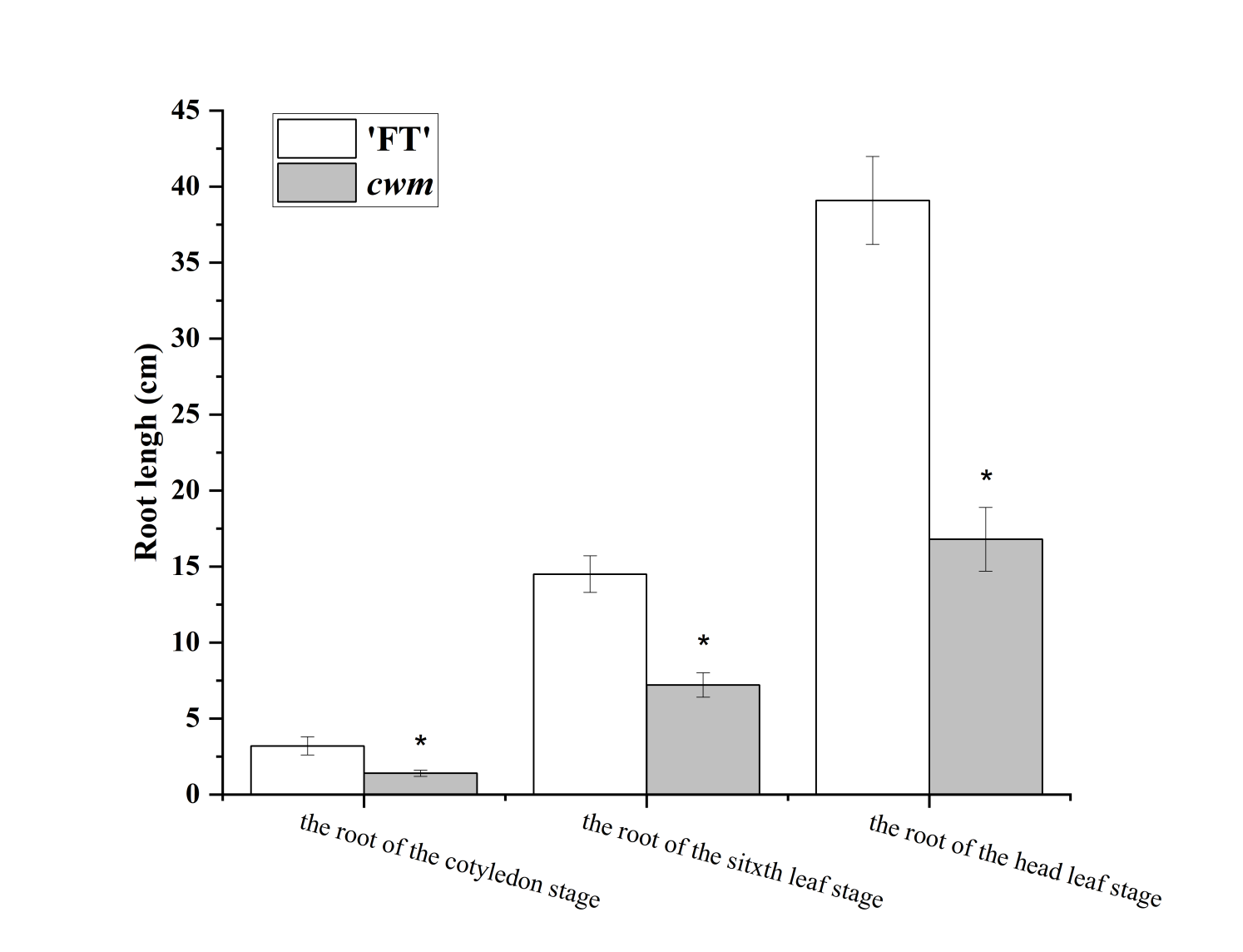


**Supplementary Figure S4.** The length determination of roots in different stages in wild-type ‘FT’ and mutant *cwm*. Asterisks represent significant differences (t test, P < 0.05).


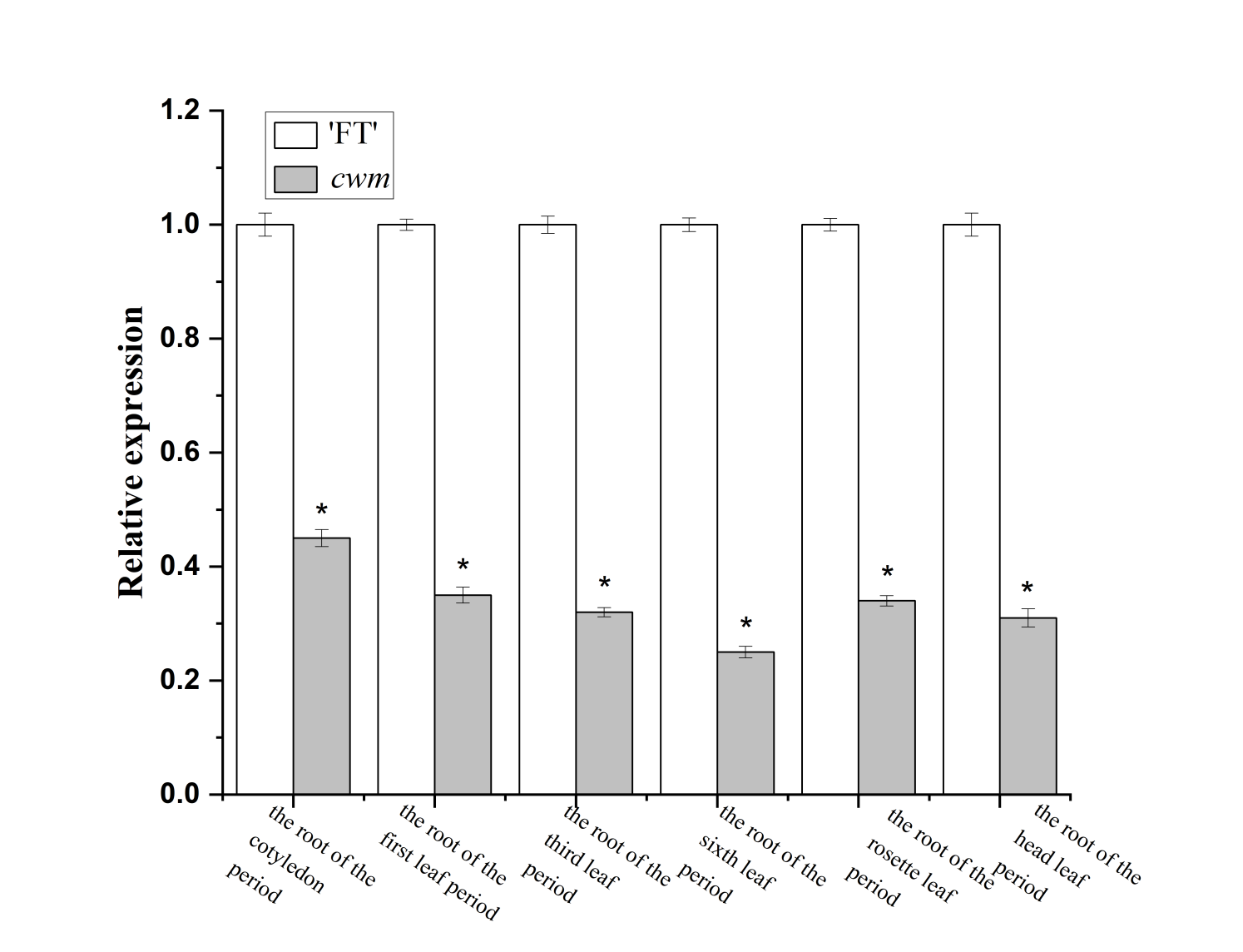


**Supplementary Figure S5.** Expression analysis of *BraA07g021970.3C* in root with different periods by qRT-PCR. The root of cotyledon, first true leaf, third true leaf, sixth true leaf, rosette leaf and head leaf were conducted. Asterisks indicate significant difference between the *cwm* and ‘FT’ (t test, P < 0.05).

**Supplementary Table S1.** Primer sequences of SSR and Indel markers

| **Markers** | **Forward primer (5'-3')** | **Reverse primer (5'-3')** | **Tm (℃)** |
| --- | --- | --- | --- |
| SSR2227 | ATCGCCATTAGATTGCCACT | GCTGACCTTTCTTACCGCTC | 56 |
| SSR2345 | GAAAAAGTAGAATAGAACTGTCGTTTT | AGATTTACACCAATAGTACATCATGAG | 56 |
| SSR3112 | GAAGAAACTCGGTGGGGAGT | AAAGAGTTCCGAAAATGGGC | 56 |
| SSR3232 | CCATGTAACCCTTTTGGATGTT | CCATGTAACCCTTTTGGATGTT | 56 |
| SSR2404 | CTGAAAATAAGGTAAAAGTCACTAAAGT | AAAAAGACAAAGATTCCTTACTGTACTA | 56 |
| SSR3105 | GTACGAACGACGAGCGTAGC | TGCTCACGGTGGAATCAAAT | 56 |
| SSR3112 | GAAGAAACTCGGTGGGGAGT | AAAGAGTTCCGAAAATGGGC | 56 |
| Indel 12 | GCTGAAGCCAGAGTCAATGAA | AGGGAAAAGGGTTACAAGGAG | 56 |
| Indel 21 | TTCTTCATTGATGTGTTATCTCCAG | TCCTTTGATTTGGTTTTATTATGTT | 56 |

**Supplementary Table S2.** Primer sequences used for cloning

| **Markers** | **Forward primer (5'-3')** | **Reverse primer (5'-3')** | **Tm (℃)** |
| --- | --- | --- | --- |
| C-1 | ATGGACGATTACACCAGAGAGATGATG | TCAATCCCTCCCATCTTCTTCATTTGC | 57 |

**Supplementary Table S3.** Primer sequences used for qRT-PCR

| **Markers** | **Forward primer (5'-3')** | **Reverse primer (5'-3')** | **Tm (℃)** |
| --- | --- | --- | --- |
| RT-1 | AGAAGAAGGGTGTCCTCGCTAA | ATGGTTGCTGCTAAAGTCGCT | 56 |

**Supplementary Table S4.** Primer sequences used for vector construction

| **Markers** | **Forward primer (5'-3')** | **Reverse primer (5'-3')** | **Tm (℃)** |
| --- | --- | --- | --- |
| V-1 | GAGAGAACACGGGGGACTTTGCAACATGGACGATTACACCAGAGAGATGATGG | CACTCCCTGAAGCGGCCGCTGTACAATCCCTCCCATCTTCTTCATTTGCTCC | 50 |

**Supplementary Table S5.** Identification of agronomic characters at heading stage

| Characteristics | ‘FT’ | *cwm* |
| --- | --- | --- |
| Mean head weight (kg) | 0.29 ± 0.05 | 0.09 ± 0.00* |
| Mean head length (cm) | 10.01± 0.56 | 7.01± 0.61* |
| Mean head width (cm) | 9.67± 0.79 | 4.67 ± 0.58* |
| Mean head length/head width ratio | 1.04 ± 0.07 | 1.50± 0.05* |

Note: Asterisks represent significant differences (t test, P < 0.05).

**Supplementary Table S6.** Primer sequences used for the identification of the transgenic lines

| **Markers** | **Forward primer (5'-3')** | **Reverse primer (5'-3')** | **Tm (℃)** |
| --- | --- | --- | --- |
| W-1 | GAAGGGTGTCCTCGCTAAGA | GAAGATTTTCAAGTGCCGCT | 57 |
